# Extracellular Vesicles in Chagas Disease: A New Passenger for an Old Disease

**DOI:** 10.3389/fmicb.2018.01190

**Published:** 2018-06-01

**Authors:** Luis M. de Pablos Torró, Lissette Retana Moreira, Antonio Osuna

**Affiliations:** Grupo de Bioquímica y Parasitología Molecular, Departamento de Parasitología, Campus de Fuentenueva, Universidad de Granada, Granada, Spain

**Keywords:** *Leishmania* spp., *Trypanosoma brucei*, *Trypanosoma cruzi*, kinetoplastids, exosome, ectosome, microvesicle, pathogen

## Abstract

Extracellular vesicles (EVs) are small lipid vesicles released by prokaryotic and eukaryotic cells containing nucleic acids, proteins, and small metabolites essential for cellular communication. Depending on the targeted cell, EVs can act either locally or in distant tissues in a paracrine or endocrine cell signaling manner. Released EVs from virus-infected cells, bacteria, fungi, or parasites have been demonstrated to perform a pivotal role in a myriad of biochemical changes occurring in the host and pathogen, including the modulation the immune system. In the past few years, the biology of *Trypanosoma cruzi* EVs, as well as their role in innate immunity evasion, has been started to be unveiled. This review article will present findings on and provide a coherent understanding of the currently known mechanisms of action of *T. cruzi*-EVs and hypothesize the implication of these parasite components during the acute and chronic phases of Chagas disease.

## Introduction

### EVs and *T. cruzi* Life Cycle

The term extracellular vesicles (EVs) is typically used to designate a compendium of different membrane-bound entities delimited by a lipid bilayer and released to the extracellular space by any type of prokaryotic or eukaryotic cell (Kalra et al., 2016). Although the populations of these nanosized vesicles are very heterogeneous, they are usually classified according to their biogenesis and size into three different categories: exosomes (40–150 nm), microvesicles (MVs) (= ectosomes/microparticles) (100–1,000 nm) and apoptotic bodies (500–4,000 nm) (Akers et al., 2013). The exosomes are of endocytic origin and are released into the medium through the fusion of multivesicular body (MVB) formed from the endo/lysosomal system with the plasma membrane of the cell, MVs are released by budding of the plasma membrane and finally apoptotic bodies which are formed through the condensation and segregation of the nucleus and the deterioration and blebbing of the plasma membrane (Akers et al., 2013). Despite their small size, the composition of the different EVs is rather complex including a wide variety of lipids, proteins, different populations of RNAs, ssDNA, and/or metabolites. Typically, the internal volume of an exosome ranges from 20 to 90 nm^3^, which suggests that a prototypical exosome roughly contains 100 proteins and 10,000 nucleotides (Vlassov et al., 2012), a figure that should be substantially higher in larger entities such as ectosomes or apoptotic bodies.

**FIGURE 1:**
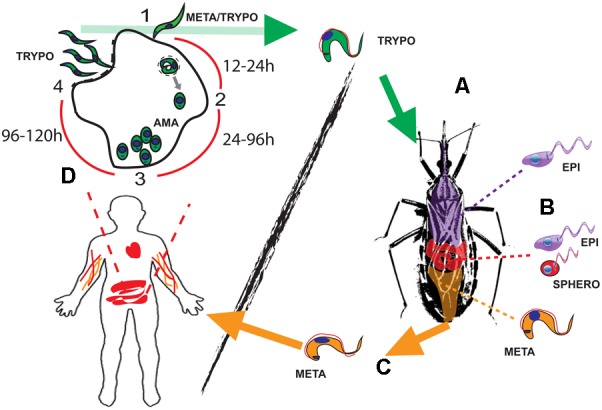
The life cycle of *Trypanosoma cruzi*. **(A)** Bloodstream trypomastigotes (green) are ingested with the bloodmeal by the reduviid vector. **(B)** Those forms will transform into epimastigotes (purple) and intermediate spheromastigotes forms (red) in the insect midgut. **(C)** New developmental differentiation into metacyclic trypomastigotes (MTs) (orange) will occur in the insect rectum before those forms are released with the feces, infecting the mammalian host. **(D)** The MTs (1st round of intracellular replication) or bloodstream trypomastigotes (green) parasites will then invade a wide range of nucleated cells in an intracellular cycle composed of up to four steps: (1) Attachment, invasion, and parasitophorous vacuole formation (0–4 h). (2) Differentiation into intracellular amastigote forms and release from parasitophorous vacuole (12–24 h). (3) Amastigote replication by binary fission (24–72 h). (4) Differentiation into bloodstream trypomastigotes and release from the infected host cell (72–120 h). The bloodstream forms are then ready to either invade new cells or been taken by the insect vector. Parasite life cycle stage abbreviations: AMA, amastigotes; META, metacyclic trypomastigotes; TRYPO, bloodstream trypomastigote; EPI, epimastigotes; SPHERO, spheromastigotes.

During the past decade, numerous studies have been conducted on EVs in the context of disease. Since EVs are virtually released by any cell type, hosts, and pathogens will be constantly shedding and sharing these products to the extra- or intracellular milieu (depending on the niche for proliferation used by the pathogen). In this context, EVs from flagellated protozoan parasites belonging to the order Kinetoplastida are being described and characterized. Kinetoplastids have monoxenous or dixenous life cycles, in which different kinetoplastid forms thrive surrounded by different cell types in the vector and the host. This large number of niches and environments creates a myriad of different EVs released, which will define parasite biodistribution, survival, and pathogenesis. Within this group of organisms, *Trypanosoma cruzi* can be found, which is the etiological agent of Chagas disease, a devastating endemic disease in 21 South and Central American countries, affecting around 6–7 million people worldwide, with 50,000 to 200,000 new cases each year (Coura et al., 2014). *T. cruzi* is an obligatory dixenous parasite, transmitted by hematophagous reduviid insects, which comprises zoonotic and anthroponotic life cycles. The life cycle of this parasite begins when hematophagous triatomine insect vectors ingest circulating bloodstream trypomastigote forms whilst having a blood meal from infected mammals such as humans (**Figure 1A**). The ingested bloodstream trypomastigotes will migrate to the insect vector midgut and differentiate into epimastigote forms, the main replicative stage, as well as into spheromastigotes, although in lower numbers (**Figure 1B**). Following attachment of epimastigotes to the wax layer of rectal cuticula, these forms will differentiate into the infective metacyclic trypomastigote (MT) forms in the insect rectum, after adhesion of the epimastigotes to the intestinal wall (**Figure 1C**). Then, the infective MTs will be released with the feces of the triatomine insect and invade epithelial and peripheral cells surrounding next to the wound (**Figure 1D**). The MT forms will transform into intracellular amastigote forms within approximately 24 h in the cytoplasm of the infected cell, giving rise to successive rounds of divisions by binary fission within 24–72 h after the infection of the host cell (Li et al., 2016) (**Figure 1D**). Once these forms occupy the entire cytoplasm of the host cell, they will differentiate into bloodstream forms (96–120 h), which will then be released from the infected cell to the bloodstream resulting in the infection of a large number of tissues. This high tissue distribution is concomitant with the onset of the “acute phase” of the disease (fatal for 2–8% of the infected subjects) lasting for approximately 2 months. This phase is usually asymptomatic including mild symptomatology (fever, headache, or muscle pain) and with large numbers of detectable circulating bloodstream trypomastigotes. This high parasitemia activates an immune response (Rassi et al., 2010), which results in the reduction of parasite numbers to very low levels (in immunocompetent hosts) and the onset of the “chronic phase” Subjects who enter this stage can either be asymptomatic which may result in a subclinical disease that lasts for decades or symptomatic with 10–40% of infected patients developing cardiomyopathies or digestive tract pathologies (Rassi et al., 2010).

As a consequence of the varying degrees of environmental stress faced, the life cycle of *T. cruzi* comprises a great variety of extracellular and intracellular forms, from highly replicative epimastigotes to low replicative intracelular amastigote forms or metabolically quiescent metacyclic and bloodstream trypomastigotes. It is thus conceivable, that kinetics, yields and composition of EVs will be extremely affected by the varying biological nature of the different developmental stages as well as the micro-/macroenvironmental physiology present in the tissues where they thrive. Given the variety of *T. cruzi* and host EVs described so far [exosomes, MVs (= ectosomes)], herein the term EVs will be used from now on for reasons of clarity unless specified.

### Could EVs Be Mediators of Local Signaling in Chagas Disease?

**FIGURE 2:**
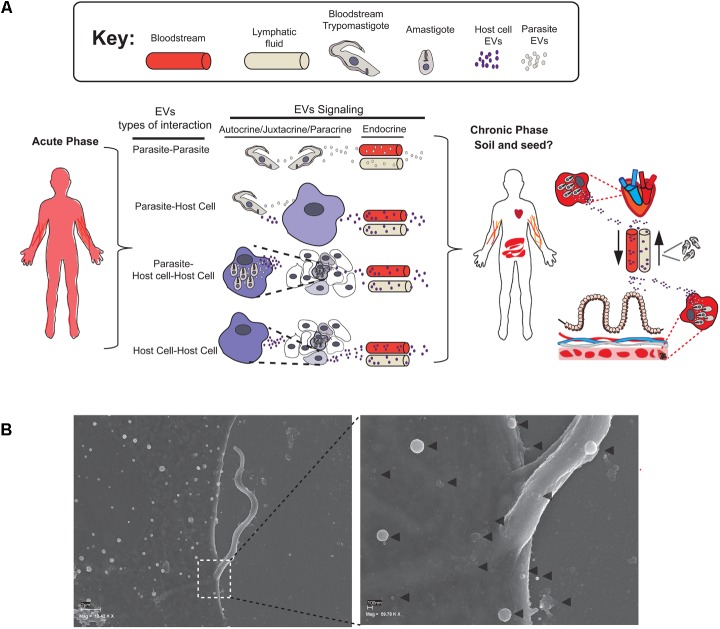
Types of EV transfer and interaction in acute and chronic phases of Chagas disease. **(A)** EVs could be released by Trypomastigote or Amastigote forms during Acute and/or Chronic phase of the disease. This secretion could be mediated between parasites, parasites and its host cells or between infected and uninfected cells mediating Autocrine, Yuxtacrine, or Paracrine type of signaling. EVs could be also released to the bloodstream or lymphatic fluids for distant endocrine targeting. During Chronic phase endocrine signaling may mediate changes for tissue re-education, colonization, and further inflammation. **(B)** Scanning electron microscopy of a MT invading a host cell. The right figure shows a magnified image of the MT point of contact with the host cell membrane. The gray arrows indicate the EVs of different sizes that may derive from both parasite and host cell. This picture corresponds to an infection of Vero Cells with MTs (strain PAN4 DTU Ia) for 2 h and belongs to the same series of scanning microscopic pictures published in Díaz Lozano et al. (2017).

Generally speaking, EV uptake and delivery is potentiated in disease conditions that are, in turn, affected by the patient’s particular status. To mediate an effect on the receptor cell, the released EVs utilize the following routes for targeting cells: endocrine (distant cells), paracrine (neighboring cells), juxtacrine (adjacent cells) or autocrine (self-targeting) signaling. Theoretically, three ways of EV transfer could occur between cells during a parasitic disease: parasite–parasite, parasite–host cell, or host cell–host cell (**Figure 2A**). These three scenarios will be clearly influenced by the phase of the disease (acute or chronic) and the patient’s status (immunodepressed, co-infections, etc.) with dynamically fluctuating levels of parasitemia and foci of infection. In the particular case of Chagas disease, the levels of circulating bloodstream trypomastigotes in pediatric patients could vary from almost 1,000 parasites/ml to 1 parasite per ml or less. The number can reach up to 1,400 parasites/ml in reactivated Chagas in patients with *T. cruzi*/HIV co-infection (Duffy et al., 2009; de Freitas et al., 2011). These figures are far below those reached in patients suffering from African sleeping sickness disease, in whom bloodstream trypomastigotes of *Trypanosoma brucei rhodesiense* replicate extracellularly reaching waves of parasitemia of more than 10^6^ organisms per milliliter (Pépin and Méda, 2001). This high yield of parasites in blood could potentially lead to a parasite–parasite paracrine EV transfer. Indeed, *T. brucei* EVs are able to transfer proteins [including virulence factors such as the serum resistance associated (SRA) protein] to recipient trypanosomes (Szempruch et al., 2016). Due to the lower yields in blood of bloodstream trypomastigotes of *T. cruzi*, it is reasonable to assume that the intracellular stages should mainly be responsible for parasite–parasite but also parasite–host cell EV transmission through paracrine and/or juxtacrine signaling, since they develop in a compartmentalized environment, increasing the likelihood of EV docking and delivery within the host cell. The ability of the amastigote stage to deliver EV protein cargoes with important effects to the host cell have been demonstrated in macrophages infected with the kinetoplastid parasite *Leishmania major* (Silverman and Reiner, 2012). This parasite is able to release via EVs virulence factors such as the metalloproteinase gp63. This release results in the activation of multiple protein-tyrosine phosphatases of the host cell implicated in the dephosphorylation of IFN-γ/Jak-STAT1 pathway and thus inhibiting the activation of macrophage microbicidal functions. Since, extracellular *T. cruzi* EVs also carry proteins belonging to the gp63 multigene family (Bayer-Santos et al., 2013), it is reasonable to think that the *T. cruzi* intracellular stage could also release this protein which would also have an important implications for the parasite intracellular survival.

The most frequently studied scenario to date is the paracrine/juxtacrine cross talk between parasite and host cell EVs (**Figure 2B**). In this context, Ramirez et al. (2017) studied MVs (= ectosomes) subpopulation of EVs from the host cell and parasite and demonstrated that the close contact between membranes results in the fusion of a bidirectional MVs (parasite–host and *vice versa*) on both the parasite and the host membranes. Moreover, the same authors demonstrated that tissue-cultured trypomastigote (TCT) MVs have the highest fusogenic potential for host cell membranes and that TCT forms have the highest rates of incorporation into the host cell, highlighting the differences among the various forms of *T. cruzi* (Ramirez et al., 2017). Cestari et al. (2012) showed that *T. cruzi* MTs and TCTs [but not epimastigote (E) forms] stimulate up to 3- to 4-fold the secretion of plasma membrane derived MVs in THP-1 monocyte cells after 60 min of exposure to the parasites (Cestari et al., 2012). Moreover, these authors also demonstrated that this host-MVs secretion is: (i) elevated independently of the *T. cruzi* strain used for infection and (ii) the secretion of host-EVs is induced in a Ca^2+^ dependent manner. This latter finding is in accordance with other cell types such as hematopoietic or neuroblastoma human cell lines (Savina et al., 2003; Emmanouilidou et al., 2010) where the increase in intra-cytosolic Ca^2+^ induce a strong ectosome-shedding response. Indeed, it has been demonstrated in different eukaryotic cell types that elevated intracellular levels of Ca^2+^ induces an asymmetric phospholipid distribution in plasma membrane by activating scramblase and flopase enzymes exposing phosphatidylserine and phosphatidylethanolamine (PS) in the outer leaflet and contributing to the activation of Ca^2+^-dependent proteases followed by ecotosome release (Yáñez-Mó et al., 2015). In this sense, the interaction of TCT forms with THP-1 monocytes leads to the increase of the PS exposure in MVs released upon this infection, being particularly increased compared to MT or E forms (Cestari et al., 2012). Accordingly, the PS exposure an subsequent release of MVs phenomena have already been described in THP-1 monocytes in exposure to different stimuli such agonists of ATP or lipopolysaccharides (LPSs) (MacKenzie et al., 2001; Liu et al., 2006; Pap et al., 2009). The higher exposure of anionic phospholipids such as PS on infected host cell EVs increases the fusogenic properties of these vesicles, which could be a pre-requisite for the exchange of information via MVs. The mobilization of Ca^2+^ from intracellular deposits has also been extensively studied during the process of *T. cruzi*-host cell invasion having several consequences for the host cell such as depolarization of host plasma membrane, F-actin depolymerization or lysosomal recruitment to the point of infection (Osuna et al., 1986; Tardieux et al., 1994; Caler et al., 2000; Scharfstein et al., 2000). Although the precise signaling cascades by which *T. cruzi* stimulates MVs and/or any other type of EV secretion in the host are still unknown, this data clearly show that the kinetics of host MV secretion is influenced by the presence of the parasite and subsequent Ca^2+^ release from intracellular deposits.

Additionally, Ramirez et al. (2017) also suggested that parasite-derived factors are capable to be revesiculated into host MVs. Although this fact still need to be experimentally demonstrated, the capacity of packing parasite proteins by host cells has been previously reported in EVs released from *Plasmodium falciparum* infected erythrocytes, enriched in *P. falciparum* membrane-associated parasite antigens (Mantel et al., 2013). In this context, early research demonstrated that paracrine/juxtacrine transfer of parasite proteins between *T. cruzi* infected host cell-to-uninfected host cells could be found in muscle, neuronal, epithelial, or fibroblast cells infected with amastigotes (Santos and Hudson, 1980; Pinho et al., 2002). Strikingly, this transfer of *T. cruzi* antigens could not be detected in lymphocyte or erythrocyte cell lines, suggesting that EV transfer is restricted to compatible cells and/or tissues (Pinho et al., 2002). In anycase, the infection of host cells with *T. cruzi* will induce the release of EVs that will have an impact in the physiological status of surrounding cells. This changes were studied by the work of Chowdhury et al. (2017) where EVs released by infected human peripheral blood mononuclear cells (PMBCs) with SylvioX10/4 strain of *T. cruzi* were isolated and incubated with THP-1 human monocites. It is worth noting that the authors used larger EVs (apoptotic bodies and/or microvesicles) for the study since the EVs purification protocol does not included ultracentrigugation steps, therefore without the exosomal fraction of EVs. After the exposure to apoptotic bodies and microvesicles, THP-1 cells were activated showing a proinflammatory gene expression profile with an increase in nitric oxide and reactive species of oxygen (ROS) (Chowdhury et al., 2017), which is in accordance with the presence of this activated immune phenotypes in chronic inflammatory pathology of Chagas disease. Therefore, strong evidence suggests that *T. cruzi* clearly influences the host-cell juxtacrine/paracrine EV release and that this situation should have a strong impact on the surrounding infected tissue.

### Could EVs Be Mediators of *T. cruzi* Tissue Biotropisms and Distant Signaling in Chagas Disease?

Due to the constitutive nature of EV secretions in cells, the presence of EVs has been detected in numerous body-fluids (nasal secretion, urine, blood, feces, breast milk, etc.) and particularly in those where *T. cruzi* proliferates (Keller et al., 2006). Under disease conditions, EV secretion has been shown to be exacerbated in the bloodstream of patients suffering from acute and chronic inflammation evoked by diseases such as sepsis, stroke, preeclampsia, atherosclerosis, diabetes mellitus, metabolic syndrome, or cancer (Yamamoto et al., 2016). This increase in circulating EVs has also been demonstrated during infection with *Plasmodium* spp., and their plasma levels associated with the severity of the disease (Mantel and Marti, 2014). Accordingly, *in vivo* infection with *T. cruzi* showed a rapid increase of EVs levels in mouse plasma (Cestari et al., 2012), which suggests a regulated secretion triggered and or increased by external cues. This increase and changes in the kinetics of host EV secretion give must be intuitively guided by factors such as the initial parasite inoculum [i.e., 50 MTs are sufficient to detect an immune response in immunocompetent mice (Mendes et al., 2013)] or the stage of the disease (acute or chronic). In this context, *in vitro* experiments have shown that EV secretion is stimulated by different stress factors such as heat, hypoxia, and/or irradiation (Yu et al., 2006; Díaz Lozano et al., 2017; Zheng et al., 2017). For instance, human non-small cell lung cancer lines treated with γ-radiation resulted in an increase of exosome release regulated by the activation of the tumor suppressor p53 oncogene (Yu et al., 2006). This fact was also observed during the course of *T. cruzi* infection in experimental infections of CD1 mice with the Brazil strain of parasites, showing that the p53 protein is upregulated in the liver 45 days post-infection, which might result in an elevated secretion of liver EVs into the bloodstream of patients with acute Chagas disease (Bouzahzah et al., 2006). These findings contrast with the transcriptome analysis of experimental *in vitro* infections of human foreskin fibroblast with the Y strain of *T. cruzi* not resulting in an increase of p53 mRNA (Li et al., 2016). Therefore and due to the distinct nature of EV populations and multifactorial responses that will trigger the release of EVs, future research must carefully analyze the possible relation between the p53 pathway and EVs secretion in the context of Chagas disease.

Variations in Chagas disease presentation, progression, and differential organ involvement are influenced by both host and parasite factors (Dutra and Gollob, 2008). The phenotypic analysis of large size circulating EVs (apoptotic bodies and microvesicles) in chronic Chagasic patients and mice chronically infected with *T. cruzi* showed that <2% of the vesicles come from platelet or cardiomyocyte, being the majority of those vesicles from monocyte/macrophage (CD14+), endothelial (CD62+) and CD4+/CD8+ T lymphocytes (Chowdhury et al., 2017). To date, there is little information on the implication of *T. cruzi* EVs in endocrine signaling and its link to tissue tropisms has, so far, neither been correlated. The probability of *T. cruzi* EV docking and targeting distant cells would mainly depend on the cell microenvironment of the infected tissue and its distance to the appropriate transport media (i.e., blood and lymphatic fluid). Different studies, have shown that the time for clearance of purified and labeled EVs from different cell types artificially introduced into the bloodstream range between a short half-life of 2 min for EVs from CD169+ cells (leukocytes and stromal cells) (Saunderson et al., 2014) to 20 min in human embryonic kidney cells (Saunderson et al., 2014) and to 5.5 h for platelet EVs (Rank et al., 2011). The values reached in the case of lymphatic fluid reflect extraordinary speed, i.e., exosomes could be distributed into lymphatic nodes within 5 min after injection into the tip of a mouse tail (Srinivasan et al., 2016). Once cleared from the bloodstream or lymphatic fluid, labeled EVs show a wide endocrine signaling distribution in a plethora of tissues where they will be cleared with different kinetics. In this sense, a great deal of research has demonstrated the relation of exosomes with the “soil and seed” hypothesis in cancer disease (Ali and Arbab, 2014). These reports have shown that during a cancerous process, endocrine signaling and metastasis formation is induced by the secretion of exosomes derived from tumors (EDTs). For instance, EDTs secreted by pancreatic cancer cells target Kupffer cells that prepare the premetastatic niche in the liver by secretion of TGF-β and subsequent mobilization of bone marrow derived cells (BMDCs) together with an increase in the production of fibronectin that generates the necessary conditions for liver metastasis (Costa-Silva et al., 2015). This process of generation of premetastatic niches by EDTs has also been recently described in the case of melanoma or breast cancer (Hoshino et al., 2015; García-Silva and Peinado, 2016). Furthermore, the injection with EDTs of breast cancer cell sub-lines with a high colonization capacity is capable of reeducating and activating cell lines marked with luciferase with low migratory capacity (Hoshino et al., 2015).

In this sense, it is conceivable that the “soil and seed” role of EDTs could have its analogy in diseases caused by pathogens such as *T. cruzi* (**Figure 2A**). First hints of this possible *T. cruzi* EVs endocrine signaling has been provided by Trocoli Torrecilhas et al. (2009). The injection of 5 μg EVs from trypomastigotes of *T. cruzi* (Y strain) into Balb/c mice 7 days prior to infection with the parasite led to substantially increased virulence of the infection (100% mortality, 22 days post-infection), with an increase in amastigote nests in heart sections. Moreover, recent work from Chowdhury et al. (2017) have shown that circulating host EVs (apoptotic bodies and/or microvesicles) isolated from blood of Chronic chagasic patients are capable to trigger an increase in IL-1β, IL-7 and IFN- γ cytoquines, ROS and NO production in macrophages, fact that was specially exacerbated in seropositive individuals who exhibited clinical disease when compared to seropositive chronic individuals with no clinical symptoms (Chowdhury et al., 2017). These EVs have been shown to be of monocyte/macrophage, endothelial and CD4+/CD8+ T lymphocytes, therefore, circulating host EVs will contribute to the inflammatory response observed in individuals with chronic Chagas disease via endocrine signaling. This possible endocrine effect of *T. cruzi* and host EVs during the disease could be related to: (i) the direct effect of EVs on target tissue via endocrine signaling, (ii) paracrine/endocrine immune cell activation (see below) and subsequent signaling. Bioluminescence imaging of chronic infections has shown that tissue damage is linked to a tenacious persistence of *T. cruzi* in tissues. Lewis et al. (2014) showed that (at least for the CL-Brener strain) the colon and stomach are permanent parasite reservoirs for the disease, where parasites are found in discrete foci of infection (Lewis et al., 2014). Surprisingly, infection in hearts was rather sporadic and non-persistent, showing a bioluminescence ranging from 10 to 80% of the animals depending on the strain (Lewis et al., 2014). More strikingly, the same authors found that while the gut was continuously infected, the foci were highly spatially dynamic, with changes in luminescence of less than 24 h. The most parsimonious interpretation is that infected phagocytes could be circulating to and from peripheral sites. However, another possible explanation could be related to a vehiculization of the firefly luciferase protein ectopically expressed on the bioluminescent parasites into EVs and further release to surrounding or distant locations from the foci of infection in a paracrine/endocrine manner. Indeed, the integration into EVs of ectopically expressed cytosolic fluorescence markers such as GFP was observed by Bayer Santos in EVs (Bayer-Santos et al., 2013), making this scenario highly probable. In this respect, further studies to clarify the role of EVs in tissue tropisms and distant signaling, and the implications for disease pathogenesis will be needed.

### Specific and Non-specific *T. cruzi* EV Protein Cargoes

**FIGURE 3:**
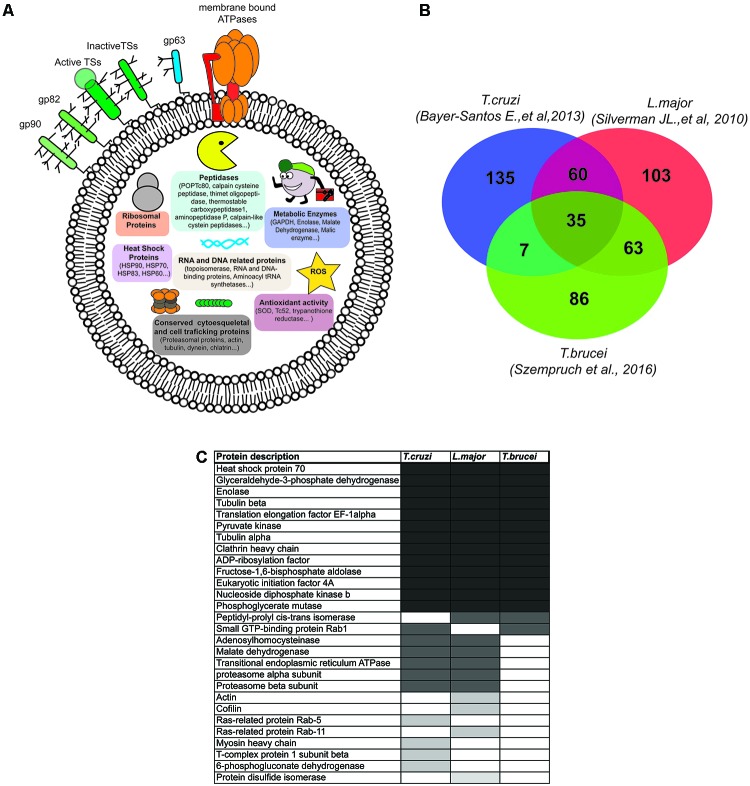
*Trypanosoma cruzi* EV protein composition. **(A)** Schematic representation of protein components described in *T. cruzi* EVs. Note that for the purpose of the figure, the different components were not represented in the scale or density of molecules. Only representative proteins from each protein group (surface, peptidases, metabolic enzymes, conserved cytosqueletal, antioxidant, RNA and DNA related proteins) are represented. **(B)** Venn diagram of *T. cruzi* (*n* = 237) (Bayer-Santos et al., 2013), *Leishmania major* (*n* = 261) (Silverman et al., 2010), and *Trypanosoma brucei* (*n* = 191) (Szempruch et al., 2016) EVs proteomic datasets (hypothetical proteins has been excluded from the analysis). **(C)** List of *T. cruzi*, *L. major*, and *T. brucei* EV proteins also found among the top 100 most frequently found vesicular proteins in mammalian EVs (Choi et al., 2013).

Proteomics has revealed the complex composition of EVs in any cell type (Choi et al., 2015). Whether or not vesicular cargo sorting and vehiculization of selected intracellular proteins in EVs is a fine-tuned regulated process or a simple constitutive release stimulated by types of stress, these components are not passive actors once released from the cell as well as necessary for cell-to-cell communication. To date, the best proteomic description of *T. cruzi* EVs has been provided by Bayer-Santos et al. (2013). The authors purified and analyzed the proteomic composition of three EV populations: ectosome, exosome (of 143 and 87 nm mean diameter, respectively) and vesicle-free fractions of *T. cruzi* epimastigote and MT forms. Overall, the comparison of E and MT EVs showed a consistent core of 70.4% (243/34) proteins shared in both stages. Only 35.8% of the EV protein composition was stage-specific (29.5% E-specific and 6.44% MT-specific respectively) confirming that independently of the stage, a large proportion of the *T. cruzi* secretome is constitutively released via EVs (Bayer-Santos et al., 2013) (**Figure 3A**). In this context, it is worth noting that EV proteins present in E and MT stages should not be discarded as potential virulence factors but rather be considered as released proteins that would play a different role and have different targets in the E (extracellular, insect vector) and MT (extra/intracellular, mammalian host) niches inhabited by parasites. For instance, it has been demonstrated that surface GPI-anchored members of the *trans*-sialidase family of genes gp82 and gp35/50, which are required for the parasite’s ability for invading the mammalian host cells, are also released in epimastigote EVs, supporting the idea of the dual role in the different stages of the life cycle (De Pablos and Osuna, 2012). Changes in the localization of parasite proteins delivered in EVs might modify their biological activity, which would make the EV protein content either multifunctional or able to switch between targeting molecules (host and parasite and *vice versa*). Moreover, different post-translational modifications (PTMs) in the E/M shared EVs core of proteins might dictate changes in vesicle localization, protein targets, and/or biological functions. Indeed, 96 out the top 100 proteins mostly identified in mammalian exosomes were reported to carry PTMs such as phosphorylation, acetylation, ubiquitination, or glycosylation which would act in different combinatorial ways to alter the protein properties within EVs (Rosa-Fernandes et al., 2017).

Proteomic profiles of EVs are highly dependent on how EVs are isolated (Yáñez-Mó et al., 2015). Different methodologies could result in difficulties for extrapolation and comparisons among distinct cell types. To date, all proteomic analyses performed in kinetoplastids used differential ultracentrifugation as the method of choice, thus variations among these analyses could be due to the varying biological properties of the different parasite kinetoplastid forms (translational activity, metabolic status, cell density, etc.) or culture conditions including pH, temperature or CO_2_.

The comparison of *T. cruzi* MT EVs (either ectosomes or exosomes) with *L. donovani* stationary-phase promastigote exosomes (30–70 nm diameter; *n* = 261) (Silverman et al., 2010) and *T. brucei* bloodstream nanotubule derived-EVs (median average of 80 nm diameter with a minimum population of 165 nm size diameter; *n* = 155) (Szempruch et al., 2016) led to a total of 35 shared proteins (**Figure 3B** and **Supplementary Data Sheet S1**). Within this common kinetoplastid EV core of proteins, 37.1% (13/35) were also found in the top 100 vesicular proteins most frequently found in mammalian EVs (Choi et al., 2013) (**Figure 3C**). In the particular case of *T. cruzi*, 28 proteins where also found in this mammalian dataset, which shows that *T. cruzi* shares some commonalities in secreted proteins via EVs with higher eukaryotes such as *Rattus norvegicus, Homo sapiens, Bos taurus* (Choi et al., 2013). Interestingly, *T. cruzi* shares more proteins with *L. major* (60) than *T. brucei* (7), which may be explained by higher commonalities between *L. major* stationary phase promastigote forms (enriched in metacyclic promastigotes) and MT forms of *T. cruzi* (De Pablos et al., 2016b), with both stages being non-replicative quiescent stages and able to invade host cells. However, this later assumption may be difficult to interprete as, in turn, *L. major* shares in turn 63 proteins with an extracellular replicative stage such as the bloodstream forms of *T. brucei* (**Figure 3B**).

The genomes of these three parasites have a total of 6158 syntenic genes, which is approximately half of the *T. cruzi* gene content (Kissinger, 2006). Conversely to the large proportion of the core genome shared by kinetoplastid parasites, proteomic analyses of EVs performed so far have revealed more differences than commonalities among these parasites, with 56.7% (135/238) of *T. cruzi* EV proteins exclusively found in this parasite. Within this subset of *T. cruzi*-EV cargoes, *T. cruzi* contains numerous members of multigenic families of GPI-anchored surface proteins such as *trans*-sialidases (TS), gp63, mucins, or mucin-associated surface proteins (MASPs) that are usually exposed in the outer leaflet of the plasma membrane (De Pablos and Osuna, 2012). Western blot, immunoelectron microscopy, and proteomic analysis have demonstrated the presence of these surface molecules in E, MT as well as TCT EVs (Bayer-Santos et al., 2013; De Pablos et al., 2016a; Lantos et al., 2016; Díaz Lozano et al., 2017). Interestingly, TSs are preferentially encapsulated in parasite-derived microvesicles (= ectosomes), while its soluble form could be cleaved of its GPI-anchor via phosphatidylinsositol-phopholipase-C (PI-PLC) (Lantos et al., 2016). This group of TSs proteins has an essential enzymatic role for *T. cruzi*, catalyzing the enzymatic transfer of syalic acid from host glycoconjugates to parasite mucin residues. This constant removal of syalic acid from host cells has important pathophysiological implications during the acute phase of the disease such as thrombocytopenia or apoptosis of immune cells (Tribulatti et al., 2005). Since *T. cruzi* non-cleaved TS shedding is preferentially routed via EVs, it is conceivable that functional molecules delivered in EVs may be more active than their soluble form (De Pablos and Osuna, 2012; Lantos et al., 2016).

Additionally, *T. cruzi* EVs also carry a wide range of potential virulence factors such as peptidases (calpain cysteine peptidase, thimet oligopeptidase, thermostable carboxypeptidase 1, or aminopeptidase P), in charge of the proteolysis of different peptide substrates (Alvarez et al., 2012) (**Figure 3A**). In this respect, proteolytically active peptidases in EVs may target proteins contained in the EVs or inside the host cell (Yáñez-Mó et al., 2015).

Finally, proteomic studies have also shown that almost all ribosomal subunit proteins were those present in *T. cruzi* tissue cultured bloodstream trypomastigotes (TCT) (unpublished results), *L. donovani* stationary phase promastigotes (Silverman et al., 2010) or *T. brucei* bloodstream EVs (Szempruch et al., 2016) (**Figure 3A**). Since the volume of an exosome is comparable to the size of the ribosomal complex (4.2–380 YL) (Vlassov et al., 2012), it may be difficult for intact ribosomal complexes to be vehiculizated into exosomes, but rather included in bigger vesicular compartments such as ectosomes or apoptotic bodies, which could function for the transfer of these cell components. This fact prompts fundamental questions that should be studied in depth such as: what is the functionality of parasite ribosomal proteins contained in EVs? Or what is the potential for translation of parasite mRNAs contained in EVs and the relevance of these products inside the host cell? In this sense, proteomic studies have prepared the ground for further dissection and study of the functionality and way of action of the EV protein content.

### New Passengers: EV-Derived Immune Complexes (EV-ICs) and Immune Response Against *T. cruzi* EVs

**FIGURE 4:**
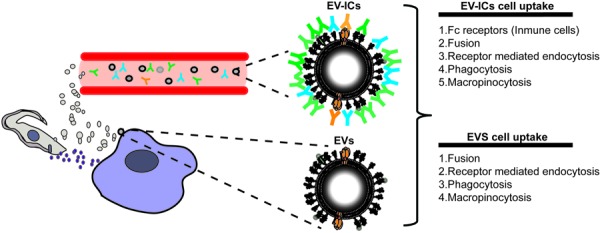
The new passengers. EVs could either target surrounding cells (EVs) or be released to the cell body fluids where they will be targeted by antibodies forming EV-ICs, which will influence the mechanisms of uptake into the targeted cells, specially by immune cells expressing Fc-gamma receptors on their surfaces.

Immune cells are one of the main targets of EVs, especially in the context of infectious diseases. Released EVs from virus-infected cells, bacteria, fungi, or parasites have been demonstrated to have a pivotal role in the modulation of the immune system. Therefore, EVs secreted during acute and/or chronic *T. cruzi* infection, should also play a role in the dissemination and survival of this parasite in the mammalian host. Indeed, several types of EVs of different organisms have already been described as promoters of the innate and acquired immune response and defined as types of PAMPs (pathogen-associated molecular patterns) (Schorey et al., 2015). Broadly, PAMPs could be formed by a wide range of molecules (lipids, proteins, carbohydrates, or nucleic acids) and are recognized by pattern recognition receptors (PPRs) (such as TLRs) present in leukocytes and various non-immune cells, which will in turn initiate a signaling cascade that leads to the activation of an immune response against the pathogen (Schorey et al., 2015). Several PAMPs have already been described for *T. cruzi*, for instance, parasite CpG-DNA released from lysed intracellular parasites stimulates TLR7 and 9 activation and production of Th1 proinflammatory cytokines or parasite α-Gal-containing glycoconjugates (such as mucins or gp85/TS) recognized by TLR2/6 leading to TNF-α production in macrophages and inhibition of IL-12 in dendritic cells (Rodrigues et al., 2012; Gravina et al., 2013). Accordingly, studies on *T. cruzi* EVs have shown that these vesicles could act as an agonist of TLR2 signaling, which leads to the secretion of proinflammatory cytokines (TNF-α and IL-6) and nitric oxide (Nogueira et al., 2015). This fact could be partially explained by the presence of GPI-anchored molecules (mucins, MASPs, or TSs) on the EV surface (see above). However, it has previously been demonstrated that *T. cruzi*-PAMPs are poorly detected by the innate immune system right at the onset of the disease, delaying the activation of the immune response most probably after the initial round of exit (approximately after the 4th to 5th day post-infection) from infected host cells, thus delaying the development of a protective immune response. In relation to the parasite and its secreted EV products, this delay may be explained by several factors: (i) the parasite invades tissues rather than immune cells immediately after the initial infection, with poor migration to surrounding tissues or draining lymph nodes (Cardoso et al., 2016), (ii) the initial inoculum and/or slow growth kinetics of the amastigote stage (slower than virus or bacteria) might, in turn, influence the minimum threshold for dendritic cell and/or phagocyte PPRs activation by EVs *in vivo*, (iii) the polyclonal B cell activation and hypergammaglobulinemia shown during the early phase of the disease will delay a parasite specific antibody response (Minoprio et al., 1988). In this sense, it is only in the third week of acute infection when parasite-specific IgGs (dominated by the IgG2a isotype) are detected in mice infected with *T. cruzi* (Bermejo et al., 2011). Therefore, IgG antibodies should poorly target EV antigens during the first 3 weeks of the disease, which could be recognized from the third week onward, when parasite-specific antibodies are raised in the serum of an infected host. Thereafter, the parasite disappears from the blood and establishes a chronic infection. With these observations, it is reasonable to think that these differences in antibody response might create two different populations of EVs during the disease, antibody-free EVs and antibody-coated EVs, which are synonymous for EV-derived circulating immune complexes (EV-ICs), whose presence have been demonstrated by some of us (Díaz Lozano et al., 2017) (**Figure 4**). During adaptive immune responses, immune complexes form when antibodies interact with their specific epitopes on soluble antigens. Immunoglobulin G (IgG) immune complexes target Fc-gamma receptors on immune cells such as dendritic cells to shuttle exogenous antigens efficiently into the cross-presentation pathway (Platzer et al., 2014). This receptor-mediated cross-presentation pathway is a well-described route for the induction of a strong CD8^+^ T cell response (Platzer et al., 2014). Using MASP conserved regions as an antigen source, it has been demonstrated that circulating parasite EVs are targeted by the immune system to form EV-ICs in chronic chagasic patients, especially in those associated with digestive pathologies (Díaz Lozano et al., 2017). In accordance, proteomic profiling of circulating immune complexes identified up to 39 antigens that show an associated humoral immune response. Interestingly, the antibody EV-targeting in the serum of chronic phase patients has also been found in host cell-infected derived EVs (Ramirez et al., 2017). Therefore, several combinations of parasite- and/or host cell derived-EVs or EV-IC populations activating juxtacrine, paracrine, or endocrine signaling could potentially prime a differentially associated immune response. Furthermore, Cestari et al. (2012) have shown that the parasite is able to evade the action of the immune complement system mediated by depositing host-cell derived EVs on its surface, which inhibits the action of complement C3 convertase. Also, *T. cruzi* complement regulatory protein (CRP) and MASP conserved N- and C- terminal regions have been found in as part of trypomastigote secreted EV cargoes (De Pablos et al., 2016a; Díaz Lozano et al., 2017) and allow the parasite to evade the complement immune system (Norris et al., 1991). Altogether, parasite EVs could (i) contribute to immune evasion (Norris et al., 1991; Cestari et al., 2012) (ii) activate the release of proinflammatory cytoquines, modulating the host immune response and (iii) free, together with circulating EV-ICs, would play a differential role activating a variety of immune cells.

## Conclusion

The active communication between *T. cruzi* and its host is a bidirectional process in which millions of EVs from both organisms participate. The final outcome would involve specific parasite tropisms and changes in immune status of the host during the chronic phase, where EVs are constantly disseminated. As reflected in this review, to date researchers have characterized only the tip of this iceberg, which has far-reaching implications in the immune response, infectivity and thus the survival of the parasite inside its host. Thus, more studies should be performed to define the biology of this complex subcellular compartment and its physiological implications during the course of Chagas disease.

## Author Contributions

All authors listed have made a substantial, direct and intellectual contribution to the work, and approved it for publication.

## Conflict of Interest Statement

The authors declare that the research was conducted in the absence of any commercial or financial relationships that could be construed as a potential conflict of interest.

## Supplementary Material

DATA SHEET S1 Comparison of the proteins identified in *T. cruzi* (*n* = 237) (Bayer-Santos et al., 2013), *Leishmania major* (*n* = 261) (Silverman et al., 2010), and *Trypanosoma brucei* (*n* = 191) (Szempruch et al., 2016) EVs proteomic datasets (hypothetical proteins has been excluded from the analysis).
